# Targeted therapeutic mild hypercapnia after cardiac arrest: a part of the bundle of care for mitigating secondary injury after cardiac arrest

**DOI:** 10.1186/s13054-017-1825-8

**Published:** 2017-09-07

**Authors:** Romain Jouffroy, Benoît Vivien

**Affiliations:** SAMU de Paris, Service d’Anesthésie Réanimation, Hôpital Necker - Enfants Malades, Assistance Publique - Hôpitaux de Paris, and Université Paris Descartes - Paris 5, Paris, France

In the July 31 issue of *Critical Care*, Eastwood and colleagues [1] discussed the discordant results of the potential therapeutic role of mild hypercapnia in the early post-resuscitation period observed by Sekhon and colleagues [2].

These two previous studies [1, 2] underlined the importance of preventing secondary cerebral injury after cardiac arrest and the role of arterial carbon dioxide on cerebral blood flow modulation in this. As described by Adrie and colleagues [3], cardiac arrest and sepsis have physiopathological similarities, including similar therapeutic interventions. For the management of septic shock, one major prognostic factor is the early initiation of appropriate treatments [4]. As underlined by Sekhon and colleagues [2], the negative results from randomized control trials of single physiological interventions (targeted temperature management and transfusion) aiming to avoid secondary injury after cardiac arrest are probably due to pathophysiological heterogeneity. Beyond this heterogeneity, we believe that, like in sepsis management, at a population scale, the benefit of a bundle of care including targeted therapeutic mild hypercapnia will be greater than a single intervention as described by Scheer and colleagues [5]. If a bundle of care to prevent secondary cerebral injury after cardiac arrest is useful to help physicians in the early stage management of these patients, personalized physiologic resuscitation targets following this stage would probably be more beneficial after intensive care unit admission. In the intensive care unit, further investigation can be performed to personalize resuscitation but will be in vain if the early stage is not managed properly.
